# Endogenous interleukin-22 prevents cardiac rupture after myocardial infarction in mice

**DOI:** 10.1371/journal.pone.0286907

**Published:** 2023-06-15

**Authors:** Mai Yamamoto, Hideo Yasukawa, Jinya Takahashi, Shoichiro Nohara, Tomoko Sasaki, Kodai Shibao, Daiki Akagaki, Kota Okabe, Toshiyuki Yanai, Tatsuhiro Shibata, Yoshihiro Fukumoto

**Affiliations:** 1 Cardiovascular Research Institute, Kurume University, Kurume, Japan; 2 Division of Cardiovascular Medicine, Department of Internal Medicine, Kurume University School of Medicine, Kurume, Japan; Scuola Superiore Sant’Anna, ITALY

## Abstract

Myocardial infarction (MI) can result in fatal myocardial rupture or heart failure due to adverse remodeling and dysfunction of the left ventricle. Although recent studies have shown that exogenous interleukin (IL)-22 shows cardioprotective effect after MI, the pathophysiological significance of endogenous IL-22 is unknown. In this study, we investigated the role of endogenous IL-22 in a mouse model of MI. We produced MI model by permanent ligation of the left coronary artery in wild-type (WT) and IL-22 knock-out (KO) mice. The post-MI survival rate was significantly worse in IL-22KO mice than in WT mice due to a higher rate of cardiac rupture. Although IL-22KO mice exhibited a significantly greater infarct size than WT mice, there was no significant difference in left ventricular geometry or function between WT and IL-22KO mice. IL-22KO mice showed increase in infiltrating macrophages and myofibroblasts, and altered expression pattern of inflammation- and extracellular matrix (ECM)-related genes after MI. While IL-22KO mice showed no obvious changes in cardiac morphology or function before MI, expressions of matrix metalloproteinase (MMP)-2 and MMP-9 were increased, whereas that of tissue inhibitor of MMPs (TIMP)-3 was decreased in cardiac tissue. Protein expression of IL-22 receptor complex, IL-22 receptor alpha 1 (IL-22R1) and IL-10 receptor beta (IL-10RB), were increased in cardiac tissue 3 days after MI, regardless of the genotype. We propose that endogenous IL-22 plays an important role in preventing cardiac rupture after MI, possibly by regulating inflammation and ECM metabolism.

## Introduction

Myocardial infarction (MI) remains a major cause of death and morbidity in westernized society. Serious outcome of MI can occur due to myocardial rupture in the acute phase and the ventricular dysfunction following the adverse remodeling of the left ventricle in the late phase. Myocardial injury caused by MI triggers an inflammatory response, which is required to repair and heal the injured myocardium. During tissue repair in general, removal of the injured tissue by inflammatory cells triggers their phenotypic modulation toward anti-inflammatory and tissue repair properties [1]. However, an excessive inflammatory response causes further myocardial injury, which contributes to subsequent complications, such as expansive ventricular remodeling and, in an extreme case, cardiac rupture [2]. Therefore, the inflammatory response must be appropriately regulated to promote myocardial healing while limiting adverse ventricular remodeling after MI. It is essential to understand how the inflammatory response is regulated to predict the clinical course and prevent adverse remodeling after MI.

The involvement of several cytokines has been reported in the inflammatory response after MI. For example, interleukin-1β (IL-1β), -6 (IL-6), -8 (IL-8), tumor necrosis factor-alpha (TNF-α), and several chemokines have been reported to promote the adverse ventricular remodeling after MI [3, 4]. On the other hand, endogenous IL-10, a representative anti-inflammatory cytokine, has been reported to protect the myocardium from ischemia/reperfusion injury [5]. IL-22, an IL-10 family cytokine, has been reported to be involved in tissue homeostasis and inflammatory response after ischemic injury in several organs, such as the liver, kidney, and brain [6–10]. Although IL-10 and IL-22 belong to the same cytokine family and cause STAT3 activation of target cells, they have different receptors and, therefore, different target cells [11–13]. IL-10 works mainly on inflammatory cells to suppress the inflammatory response [14, 15]. In contrast, IL-22 works in certain cells, such as epithelial cells, fibroblasts, and endothelial cells that express IL-22 receptor, but not on inflammatory cells that do not express IL-22 receptor. For myocardial ischemic injury, exogenously administered IL-22 has been reported to ameliorate cardiac dysfunction and ventricular remodeling after MI via the hepatocyte-dependent mechanism [16]. We have recently reported that IL-22 administration prevents myocardial injury via STAT3 activation in cardiomyocytes [17], which can prevent the development of ventricular remodeling after MI [18–21]. However, the role of endogenous IL-22 in regulating the inflammatory response and ventricular remodeling after MI has not been clarified. In the current study, we investigated the role of endogenous IL-22 in the regulation of the inflammatory response and ventricular remodeling using a mouse model of MI.

## Methods

### Mouse model of myocardial infarction

All experimental protocols involving animal were approved by the Animal Experiments Review Boards of Kurume University (Permit Number: 2017-125-1, 2018–216, 2019–085, 2020–053, 2021–046, 2022–038). All procedures on the mice were performed under general anesthesia with isoflurane and all efforts were made to minimize suffering. The mice were maintained with normal chow and freely available drinking water. Wild-type (WT) C57BL/6j mice were purchased from Charles River Laboratories Japan. We also used IL-22 knock-out (IL-22KO) mice created as described previously [22] in the C57BL/6j background. As the incidence of MI is higher in men than in women [23], we used male mice in this study. In 8 to 12-week-old male WT and IL-22KO mice, MI was induced by permanent ligation of the left coronary artery as described previously [21]. Briefly, mice were anaesthetized with inhaled isoflurane (5% for induction; 1–2% for maintenance) administered using an endotracheal tube and positive pressure ventilation (HSE miniVent, Harvard Apparatus GmbH). Body temperature was maintained at 37°C using a heating pad and monitored with a rectal thermometer. The thoracic cavity was opened by left thoracotomy. Next, an 8–0 prolene suture was passed under the left anterior descending (LAD) coronary artery at the inferior edge of the left atrium and tied to produce an occlusion. The chest was closed with continuous 6–0 prolene sutures. The endotracheal tube was removed following resumption of spontaneous respiration. To prevent death due to hypothermia caused by circulatory failure after surgery, mice were kept overnight in cages placed on a warming plate heated to 37°C. A sham operation included all procedures, excepting the ligation of coronary artery.

### Cardiac function and hemodynamic measurement

Systolic blood pressure and heart rate were non-invasively and measured in the awake state using a tail cuff method with a MK-2000 (Muromachi Kikai, Tokyo, Japan). On the indicated days before and after MI, transthoracic ultrasound cardiography was performed using a Vevo3100 ultrasound machine (FUJIFILM VisualSonics, Toronto, Canada) as described previously [17, 21]. Mice were kept under light anesthesia using isoflurane (5% for induction; 1–2% for maintenance) with the heart rate over 450 bpm. Left ventricular internal dimension in diastole (LVID:d), left ventricular internal dimension in systole (LVID:s), interventricular septal thickness in diastole (IVS:d), and left ventricular posterior wall thickness in diastole (LVPW:d) were measured in M-mode at the level of the papillary muscle.

### Triphenyltetrazolium chloride staining

Triphenyltetrazolium chloride (TTC) staining was performed 3 days after MI. Each mouse was kept under deep anesthesia using isoflurane (5%), the heart excised, and cut into five transverse slices with equivalent thickness. These slices were incubated in 1% TTC solution (Sigma Aldrich, St. Louis, MO) at 37°C for 10 min and photographed under the microscope (Leica, M165; Wetzlar, Germany). On each image, we measured the infarct area (i.e., the area lacking TTC staining) and ventricular wall areas using Image-Pro software (version 10; Media Cybernetics, Rockville, Maryland). The ratio of the net infarct area in the net ventricular wall area was calculated from five slices.

### Western blot analysis

Whole ventricle tissues or infarcted areas of heart tissues were homogenized in lysis buffer containing 25 mM HEPES (pH 7.5), 1% Triton X 100, 150 mM NaCl, 10% glycerol, 1 mM sodium orthovanadate, 50 mM sodium fluoride, 10 mM sodium pyrophosphate, and protease inhibitor cocktail (Sigma-Aldrich, St. Louis, MO). The tissue extracts were resolved by the Nu-PAGE system (Thermofisher Scientific, Waltham, MA), and Western blot analysis performed as described previously [17, 21]. Antibodies against tyrosine-phosphorylated STAT3 (p-STAT3; #9145, 500 ng/mL) and STAT3 (#12640, rabbit polyclonal, 23 ng/mL) were purchased from Cell Signaling Technology (Danvers, MA). Antibody against glyceraldehyde 3-phosphate dehydrogenase (GAPDH; MAB374, 6C5, 200 ng/mL) was purchased from Merck Millipore (Darmstadt, Germany). Antibody against IL-22R1 (ab5984, 1 μg/mL) was purchased from Abcam (Cambridge, United Kingdom). Antibody against IL-10RB (15102-1-AP, 450ng/mL) was purchased from Proteintech (Chicago, IL).

### Histological analysis

Freshly isolated hearts were fixed in 4% paraformaldehyde (PFA), dehydrated, embedded in paraffin, and sectioned (5-μm-thick). These sections were then subjected to H&E staining, picrosirius red staining, and immunostaining. Immunostaining was performed using the VECTASTAIN ABC Mouse IgG Kit (PK-4002; Vector Laboratories, Burlingame, CA) for alpha smooth muscle actin (α-SMA; A5228, 125 ng/mL; Sigma-Aldrich), the catalyzed signal amplification (CSA) system (CSA II Biotin-free Tyramide Signal Amplification system, K1497; Agilent Technologies, Santa Clara, CA) for IL-22R1 (ab5984, 1 μg/mL; Abcam, Cambridge, United Kingdom), and the VECTOR M.O.M. Immunodetection Kit (PK-2200; Vector Laboratories, Burlingame, CA) for Iba1 (MABN92, 2 μg/mL; Merck Millipore, Burlington, MA) following the manufacturers’ protocols. Microscopic images were obtained using an automated microscope (BZ-9000, Keyence, Osaka, Japan). We defined the border area in the MI samples where cardiomyocytes with nuclei (alive) and without nuclei (dead) were mixed in a single visual field under the 20x objective lens. After taking images of the entire border area from a single tissue slice, the tissue area and positive immunostaining area were determined using the built-in analysis software of the microscope to calculate the percentage of positive area in the tissue.

### RNA extraction and real-time PCR

Total RNA was isolated using TRIzol (15596–026; Thermo Fisher Scientific, Waltham, MA) and the PureLink RNA Mini Kit (12183018A, Thermo Fisher Scientific) as described previously [17, 21]. Total RNA (1 μg) was converted into cDNA using the High-Capacity RNA-to-cDNA Kit (4387406; Thermo Fisher Scientific). Real-time polymerase chain reaction (PCR) analysis was performed to assess the gene expression using the StepOnePlus Real-Time PCR System (Thermo Fisher Scientific) as described previously [17, 21]. The expression profiles of inflammation and extracellular matrix (ECM) metabolism genes were obtained using the RT^2^ Profiler PCR array for mouse fibrosis (PAMM-120Z; Qiagen, Hilden, Germany) according to the manufacturer’s instructions. The expression of individual genes was examined by real-time PCR using the corresponding primer sets (Thermo Fisher Scientific). Primer information were shown in **Table 1**.

**Table 1:** Primer list.

Symbol	Description	Assay ID
*Col1a1*	Collagen, type I, alpha 1	Mm00801666_g1
*Col3a1*	Collagen, type III, alpha 1	Mm00802300_m1
*Gapdh*	glyceraldehyde-3-phosphate dehydrogenase	Mm99999915_g1
*Il1b*	interleukin 1β	Mm00434228_m1
*Il6*	interleukin 6	Mm00446190_m1
*Il10*	interleukin 10	Mm01288386_m1
*Il19*	interleukin 19	Mm01288324_m1
*Il20*	interleukin 20	Mm00445341_m1
*Il22*	interleukin 22	Mm01226722_g1
*Il24*	interleukin 24	Mm00474102_m1
*Mmp2*	matrix metallopeptidase 2	Mm00439498_m1
*Mmp9*	matrix metallopeptidase 9	Mm00442991_m1
*Mmp13*	matrix metallopeptidase 13	Mm00439491_m1
*Timp1*	tissue inhibitor of metalloproteinase 1	Mm01341361_m1
*Timp2*	tissue inhibitor of metalloproteinase 2	Mm00441825_m1
*Timp3*	tissue inhibitor of metalloproteinase 3	Mm00441826_m1
*Timp4*	tissue inhibitor of metalloproteinase 4	Mm01184417_m1
*Tnf*	tumor necrosis factor	Mm00446190_m1

### Microbeads-based analysis

Serum levels of IL-22 were measured before and after MI at the indicated time points using Bio-Plex system (Bio-Rad Laboratories, Hercules, CA), as previously described [17]. Serum was separated from the blood sample according to the manufacturer’s instructions and stored at −80°C until use.

### Recombinant IL-22 administration

Recombinant mouse IL-22 (210–22; Peprotech EC Ltd., Rocky Hill, NJ) was dissolved in 1% bovine serum albumin (BSA) in phosphate-buffered saline (PBS). IL-22KO mice were subcutaneously injected with recombinant mouse IL-22 (100 ng/g) or an equal volume of 1% BSA in PBS twice daily for 7 days after MI.

### Statistical analysis

Statistical analyses were performed using GraphPad PRISM 8 (GraphPad Software, San Diego, CA). For comparisons of three or more groups, one-way ANOVA was performed, followed by Dunnett’s T3 multiple comparison test when the data passed the Shapiro-Wilk normality test. Otherwise, the Kruskal-Wallis test was performed, followed by Dunn’s multiple comparison test. Comparisons between two groups were performed using Welch’s test when the data passed the Shapiro-Wilk normality test. Otherwise, the Mann-Whitney test was performed. Data are expressed as median±quartile. A P-value < 0.05 was considered significant.

## Results

### Mortality and cardiac rupture after MI

**Fig 1:**
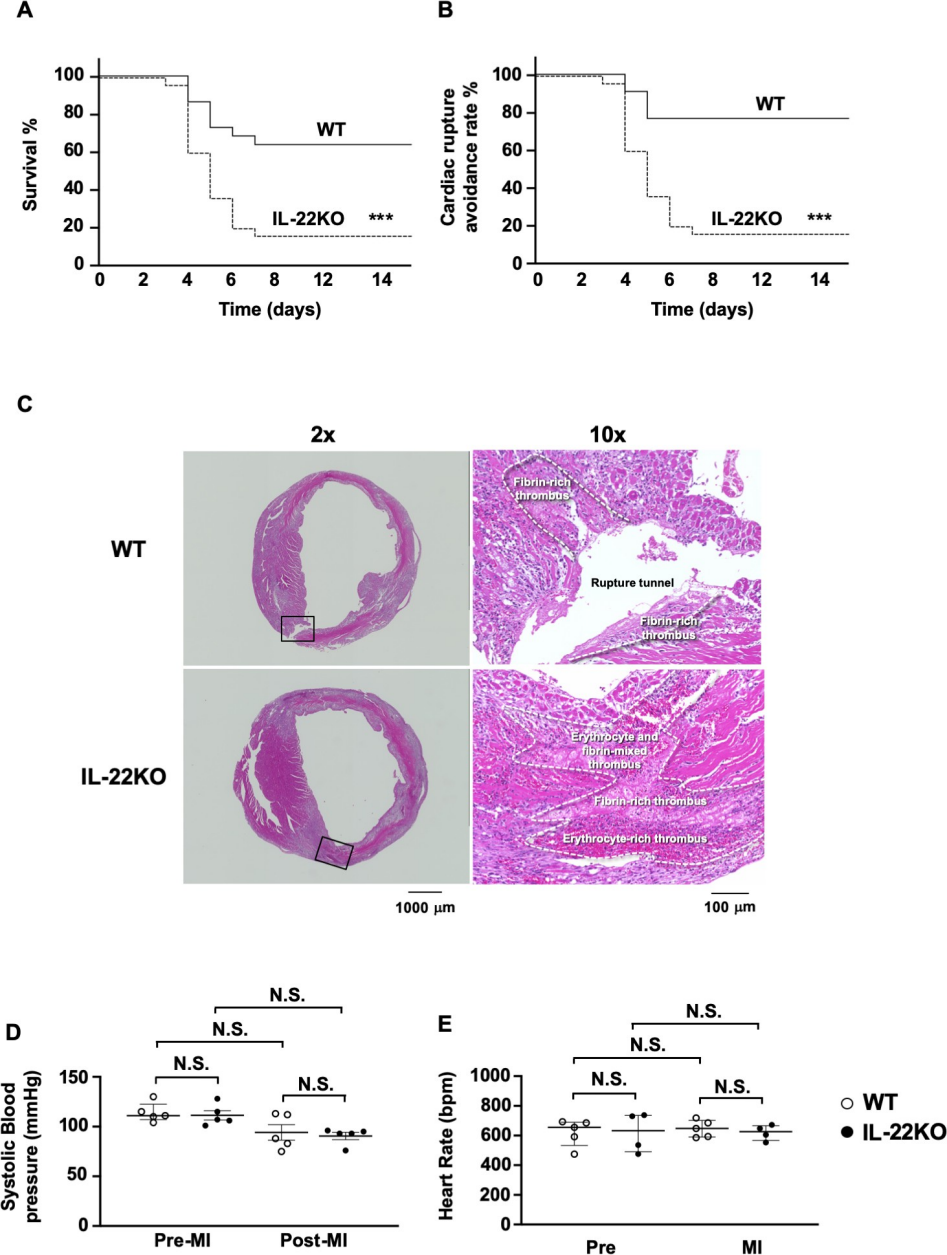
Mortality and cardiac rupture after MI in IL-22KO mice. (**A**) Survival curve and (**B**) cardiac rupture avoidance rates after MI in WT and IL-22KO mice (n = 22–25 for each group); ***P < 0.001 vs. WT mice, Log-rank (Mantel-Cox) test. (**C**) H&E staining of hearts from ruptured WT mice and IL-22KO mice. Representative photographs of hearts are shown. (**D**) Systolic blood pressure and (**E**) heart rate in WT or IL-22KO mice before MI and 3 days after MI (n = 5 for each group); N.S., non-significant, Kruskal-Wallis test/Dunn’s multiple comparison test.

First, we examined effect of IL-22 gene deletion on the survival rate after MI. Among WT mice, 22% died within the first week after MI **(Fig 1A)** compared to 85% of IL-22KO mice (P < 0.001). Among the WT mice, 11% died of fatal cardiac rupture, whereas 77% of IL-22KO mice died of cardiac rupture (**Fig 1B**, P < 0.001). In both WT and IL-22KO mice, cardiac rupture occurred between 4 and 7 days after MI. Most of the cardiac ruptures occurred in the border area between the infarct and non-infarct tissue [24, 25]. In the rupture site, fibrin-rich thrombus was observed regardless of the genotype **(Fig 1C)**. Systolic blood pressure or heart rate showed no significant changes before and after MI regardless of the genotype (**Fig 1D and 1E**).

### Left ventricular function and infarct size

**Fig 2:**
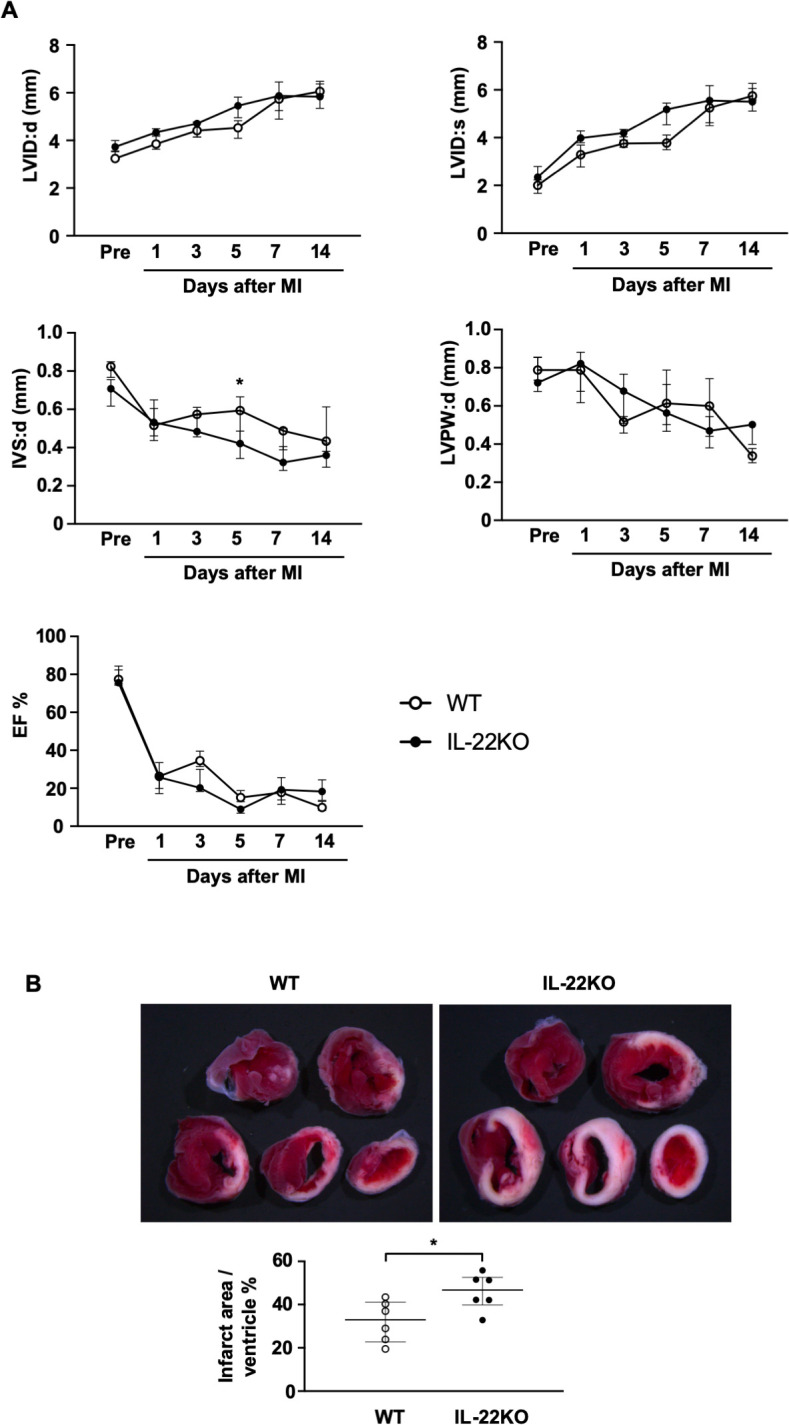
Comparison of cardiac function and infarct size after MI. (**A**) Echocardiography was performed at the indicated time points (n = 4–5 for each group); *P < 0.05 vs. respective WT mice, Kruskal-Wallis test/Dunn’s multiple comparison test. LVID:d, left ventricular internal dimension in diastole; LVID:s, left ventricular internal dimension in systole; interventricular septal thickness in diastole; IVS:d, left ventricular posterior wall thickness in diastole; LVPW:d, EF, ejection fraction. (**B**) Representative images of TTC staining in WT or IL-22 KO mice 3 days after MI. Graphs show quantification of infarct size expressed as infarct area/ventricular wall area (n = 6 for each group); *P < 0.05 vs. WT mice, Mann-Whitney test.

Next, we assessed the effect of IL-22 gene deletion on the left ventricular geometry, function and infarct size. Echocardiographic assessment showed no significant difference in LVID:d, LVID:s, LVPW:d and ejection fraction (EF) between WT mice and IL-22KO mice before or after MI (**Fig 2A**). IVS:d was significantly lower in IL-22KO than WT only on 5 days after MI, but no difference was observed on other time points after MI. TTC staining 3 days after MI showed that the infarct area was significantly larger in IL-22KO mice than in WT mice (**Fig 2B**, P < 0.05).

### Cell infiltration in myocardium after MI

**Fig 3:**
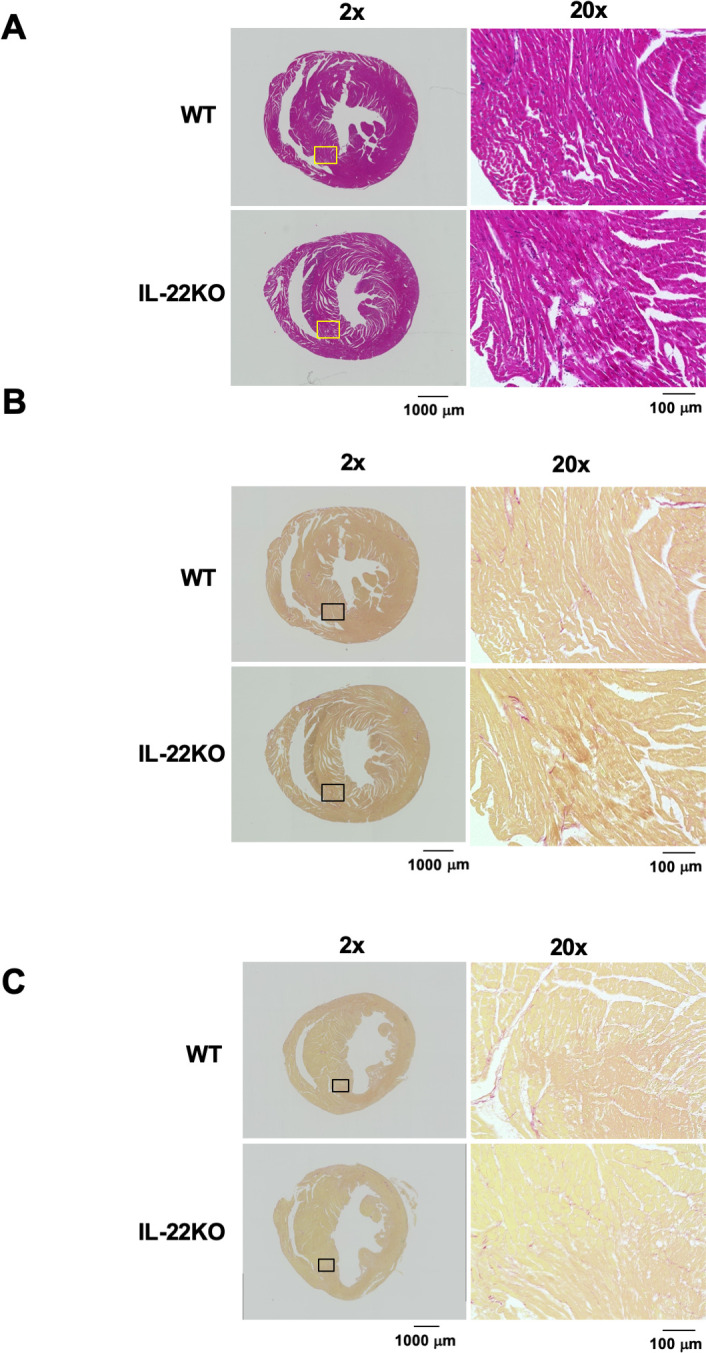
Effect of IL-22 gene deletion on histological changes before and after MI. (**A**) H&E and (**B**) picrosirius red staining of hearts before MI. Representative images are shown from 3 independent observations in each genotype. (**C**) Picrosirius red staining of hearts 3 days after MI. Representative images are shown from 6 independent observations in each genotype.

**Fig 4:**
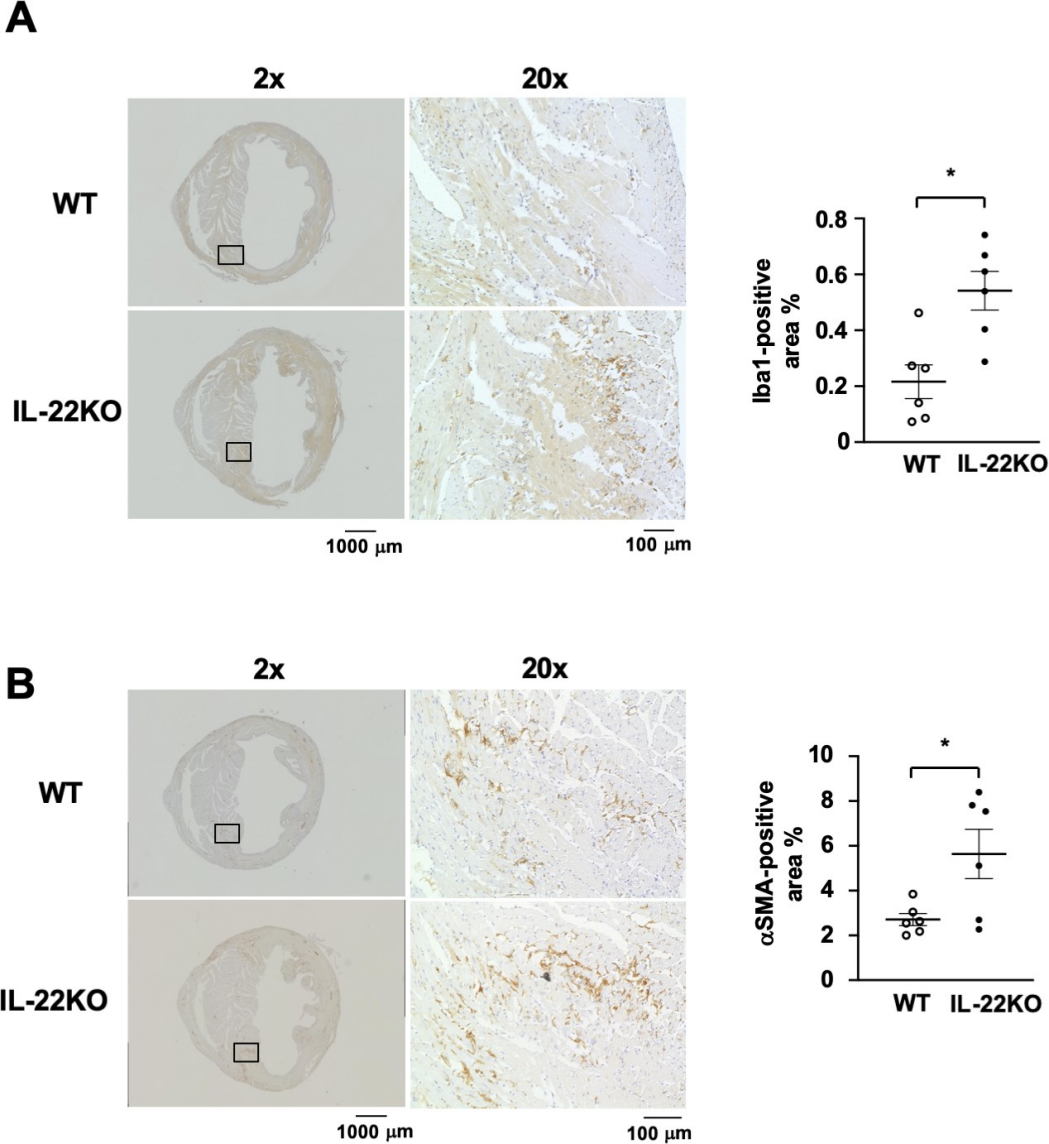
Effect of IL-22 gene deletion on cell infiltration after MI. (**A**) Immunostaining of Iba1 and (**B**) α-SMA in hearts 3 days after MI. Representative images are shown from 6 independent observations in each group. Values are expressed as the percentage of Iba1-positive or α-SMA-positive area per tissue area. n = 6 for each group; *P < 0.05 vs. WT mice, Welch’s test.

Next, we compared the histological features between WT and IL-22KO mice before and 3 days after MI, when cardiac rupture was about to occur. Compared to hearts from WT mice, the hearts from IL-22KO mice presented no gross abnormality before MI **(Fig 3A and 3B)**. Three days after MI, picrosirius red staining did not show significant collagen fiber deposition in the hearts of WT or IL-22KO mice **(Fig 3C)**. We examined the effect of IL-22 gene deletion on Iba1-positive macrophage infiltration 3 days after MI in the border area between the infarcted and non-infarcted tissue, the place prone to cardiac rupture [24, 25]. Compared to WT mice, IL-22KO mice showed significant increase in macrophage infiltration in the border area (**Fig 4A**, P < 0.05). We also examined the infiltration of α-SMA-positive myofibroblasts. Compared to the WT mice, IL-22KO mice showed significant increase in myofibroblast infiltration in the border area (**Fig 4B**, P < 0.05).

### Expression of inflammation- and ECM metabolism-related genes

**Fig 5:**
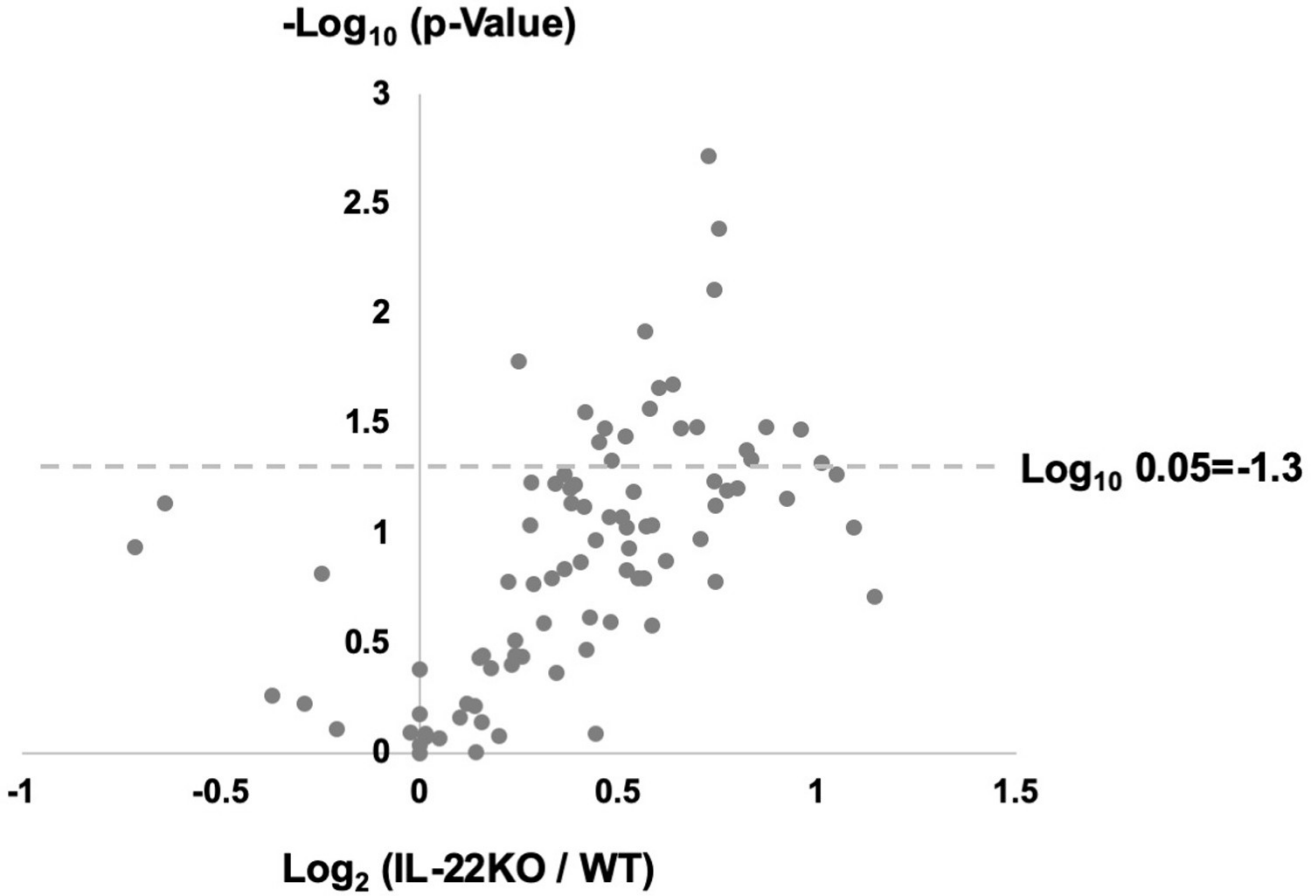
Effect of IL-22 gene deletion on the expression pattern of inflammation- and ECM metabolism-related genes. Total RNA was prepared from the infarcted heart 3 days after MI and subjected to real time PCR array analysis. Values were normalized to *Gapdh* and expressed as the fold change from the values in WT mice (n = 5 for each group). The fold change and P-values are shown in the volcano plot.

**Fig 6:**
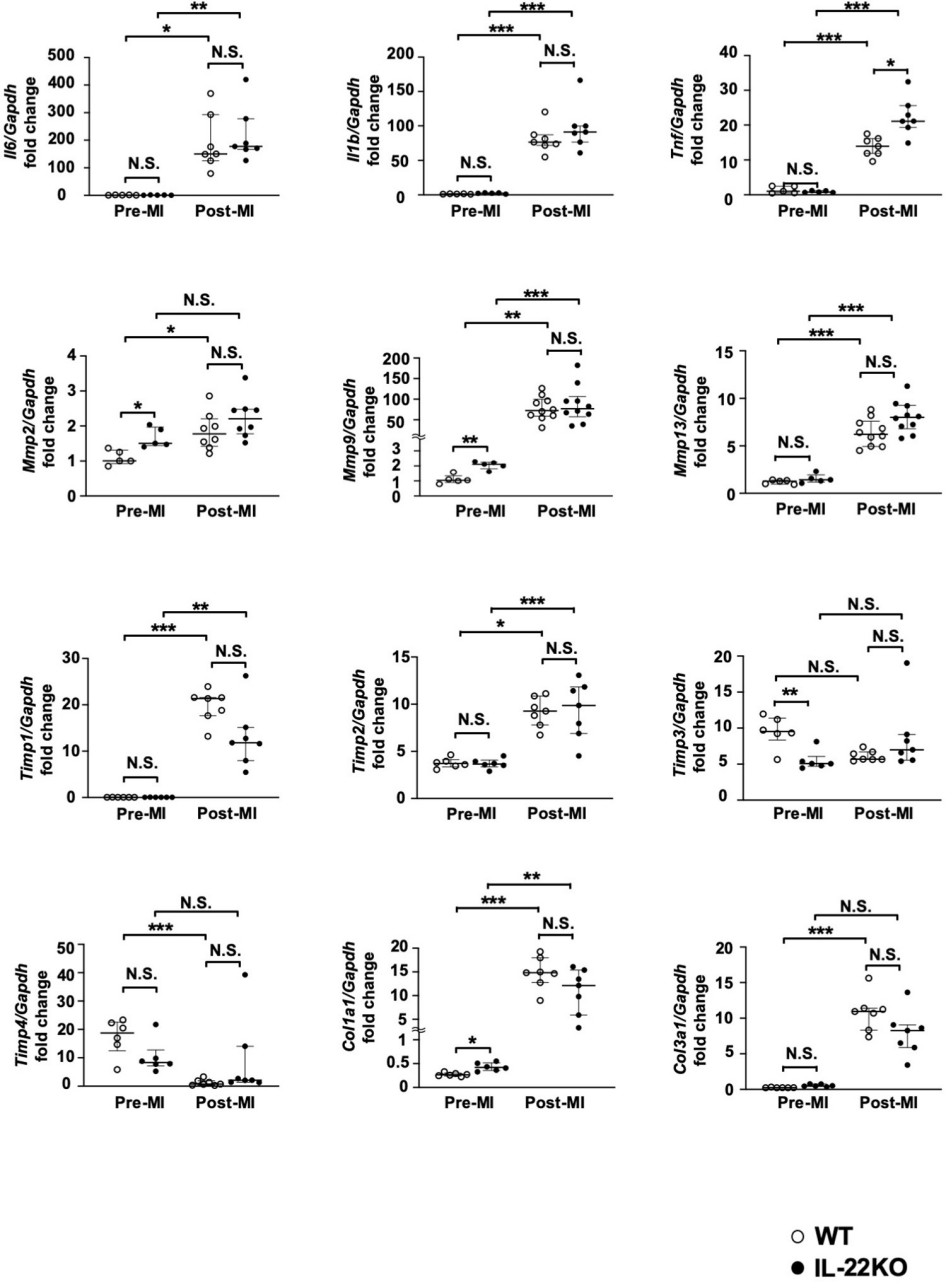
Effect of IL-22 gene deletion on the expression of inflammation- and ECM metabolism-related genes. Total RNA was prepared from whole ventricle before or 3 days after MI and subjected to real-time PCR analysis. Values are normalized to *Gapdh* and expressed as the fold change from the values in WT mice before MI (n = 5 to 10 for each group); *P < 0.05, **P < 0.01, ***P < 0.001, N.S., non-significant, one-way ANOVA/Dunnett’s T3 multiple comparison test or Kruskal-Wallis test/Dunn’s multiple comparison test.

To examine the effect of IL-22 gene deletion on the inflammatory response after MI, we measured the expression of genes involved in inflammation and ECM metabolism. We used a real-time PCR array to observe the gene expression pattern in hearts excised 3 days after MI **(Fig 5 and Table 2)**. The gene expression pattern revealed the trend for higher expression in IL-22KO mice than in WT mice after MI. To compare inflammation- and ECM metabolism-related genes, real-time PCR analysis was conducted for individual genes in the hearts before and 3 days after MI **(Fig 6)**. Before MI, IL-22KO mice showed higher expression of *Mmp2*, *Mmp9 and Col1a1* than WT mice, albeit at lower levels than those after MI. On the contrary, IL-22KO mice showed lower expression of *Timp3* than WT mice before MI. Other genes showed no significant difference between WT and IL-22KO mice before MI. After MI, *Il6*, *Il1b*, *Tnf*, *Mmp9*, *Mmp13*, *Timp1*, *Timp2* and *Col1a1* were expressed at significantly higher levels compared to before MI, regardless of the genotype. The induction of *Tnf* after MI was more prominent in IL-22KO mice than in WT mice. *Mmp2*, *Col3a1* were significantly increased after MI only in WT, whereas *Timp4* was significantly suppressed after MI in WT mice but not in IL-22KO mice, probably because the expression tended lower in IL-22KO mice than in WT mice before MI. As for the gene expression of IL-10 family cytokines other than IL-22 in the heart, *Il10*, *Il19*, and *Il24* were significantly increased after MI regardless of the genotype (**S1 Fig**). *Il20* expression was undetectable in the heart in any condition.

**Table 2:** Target genes and results of PCR array.

Symbol	Description	Fold Change	P-value
Fasl	Fas ligand (TNF superfamily, member 6)	2.24	0.192883
Stat1	Signal transducer and activator of transcription 1	2.13	0.093432
Ccl12	Chemokine (C-C motif) ligand 12	2.02	**0.047541**
Grem1	Gremlin 1	2.00	0.054049
Ccr2	Chemokine (C-C motif) receptor 2	1.94	**0.033671**
Timp2	Tissue inhibitor of metalloproteinase 2	1.90	0.069019
Smad6	MAD homolog 6 (Drosophila)	1.83	**0.032969**
Col3a1	Collagen, type III, alpha 1	1.77	**0.041927**
Il5	Interleukin 5	1.76	**0.046219**
Hgf	Hepatocyte growth factor	1.74	0.062162
Il4	Interleukin 4	1.71	0.063851
Tgfb2	Transforming growth factor, beta 2	1.69	**0.004075**
Thbs2	Thrombospondin 2	1.68	0.164844
Il10	Interleukin 10	1.67	0.074645
Mmp13	Matrix metallopeptidase 13	1.67	**0.007798**
Tgfb3	Transforming growth factor, beta 3	1.66	**0.001900**
Col1a2	Collagen, type I, alpha 2	1.63	0.105659
Itgb8	Integrin beta 8	1.62	**0.032835**
Itgb5	Integrin beta 5	1.58	**0.033012**
Jun	Jun oncogene	1.52	**0.021660**
Acta2	Actin, alpha 2, smooth muscle, aorta	1.50	**0.027137**
Cebpb	CCAAT/enhancer binding protein (C/EBP), beta	1.50	0.091200
Ifng	Interferon gamma	1.50	0.260993
Dcn	Decorin	1.49	0.092731
Tgfbr1	Transforming growth factor, beta receptor I	1.48	**0.012050**
Tnf	Tumor necrosis factor	1.48	0.159165
Tgfbr2	Transforming growth factor, beta receptor II	1.47	0.160458
Serpinh1	Serine (or cysteine) peptidase inhibitor, clade H, member 1	1.45	0.064151
Ccl3	Chemokine (C-C motif) ligand 3	1.44	0.147730
Edn1	Endothelin 1	1.44	0.116787
Itga1	Integrin alpha 1	1.44	0.093843
Ctgf	Connective tissue growth factor	1.43	**0.036106**
Tgif1	TGFB-induced factor homeobox 1	1.42	0.084324
Itgav	Integrin alpha V	1.40	**0.046293**
Mmp8	Matrix metallopeptidase 8	1.40	0.253702
Mmp14	Matrix metallopeptidase 14 (membrane-inserted)	1.39	0.084203
Nfkb1	Nuclear factor of kappa light polypeptide gene enhancer in B-cells 1, p105	1.38	**0.033274**
Eng	Endoglin	1.37	**0.038276**
Lox	Lysyl oxidase	1.36	0.107183
Snai1	Snail homolog 1 (Drosophila)	1.36	0.815019
Plau	Plasminogen activator, urokinase	1.35	0.242265
Sp1	Trans-acting transcription factor 1	1.34	**0.028021**
Thbs1	Thrombospondin 1	1.34	0.340269
Timp3	Tissue inhibitor of metalloproteinase 3	1.33	0.075628
Smad7	MAD homolog 7 (Drosophila)	1.32	0.134767
Smad2	MAD homolog 2 (Drosophila)	1.31	0.060203
Tgfb1	Transforming growth factor, beta 1	1.31	0.072445
Cav1	Caveolin 1, caveolae protein	1.30	0.061953
Pdgfb	Platelet-derived growth factor, B polypeptide	1.29	0.053758
Smad3	MAD homolog 3 (Drosophila)	1.29	0.145775
Bcl2	B-cell leukemia/lymphoma 2	1.27	0.059526
Cxcr4	Chemokine (C-X-C motif) receptor 4	1.27	0.431745
Ltbp1	Latent transforming growth factor beta binding protein 1	1.26	0.160661
Akt1	Thymoma viral proto-oncogene 1	1.24	0.256586
Inhbe	Inhibin beta E	1.23	0.307830
Itga2	Integrin alpha 2	1.22	0.169898
Itgb1	Integrin beta 1 (fibronectin receptor beta)	1.22	0.058935
Smad4	MAD homolog 4 (Drosophila)	1.21	0.091569
Il1b	Interleukin 1 beta	1.20	0.364702
Stat6	Signal transducer and activator of transcription 6	1.18	0.357968
Mmp2	Matrix metallopeptidase 2	1.17	0.393860
Plat	Plasminogen activator, tissue	1.17	0.166755
Ccl11	Chemokine (C-C motif) ligand 11	1.15	0.835752
Itgb3	Integrin beta 3	1.13	0.411546
Ilk	Integrin-linked kinase	1.12	0.357936
Il13	Interleukin 13	1.11	0.594169
Mmp1a	Matrix metallopeptidase 1a (interstitial collagenase)	1.11	0.724869
Pdgfa	Platelet-derived growth factor, alpha	1.11	0.366783
Itgb6	Integrin beta 6	1.10	0.612644
Mmp3	Matrix metallopeptidase 3	1.10	0.998504
Myc	Myelocytomatosis oncogene	1.07	0.688170
Itga3	Integrin alpha 3	1.03	0.859449
Il13ra2	Interleukin 13 receptor, alpha 2	1.01	0.853403
Mmp9	Matrix metallopeptidase 9	1.01	0.821614
Serpina1a	Serine (or cysteine) peptidase inhibitor, clade A, member 1a	1.01	0.417873
Bmp7	Bone morphogenetic protein 7	1.00	0.922870
Plg	Plasminogen	0.99	0.668809
Serpine1	Serine (or cysteine) peptidase inhibitor, clade E, member 1	0.99	0.810927
Egf	Epidermal growth factor	0.87	0.783207
Vegfa	Vascular endothelial growth factor A	0.84	0.153023
Timp1	Tissue inhibitor of metalloproteinase 1	0.82	0.596287
Timp4	Tissue inhibitor of metalloproteinase 4	0.77	0.547844
Agt	Angiotensinogen (serpin peptidase inhibitor, clade A, member 8)	0.64	0.073203
Il1a	Interleukin 1 alpha	0.61	0.114845

Fold change (2^-ΔΔCT^) is the normalized gene expression (2^-ΔCT^) in IL-22KO mice divided by the normalized gene expression (2^-ΔCT^) in WT mice. The p-values were calculated based on a Student’s t-test of the replicate 2^-ΔCT^ values for each gene in wild-type mice and IL-22 knockout mice. P-values < 0.05 are indicated in bold font.

### Expression of IL-22-related molecules after MI

**Fig 7:**
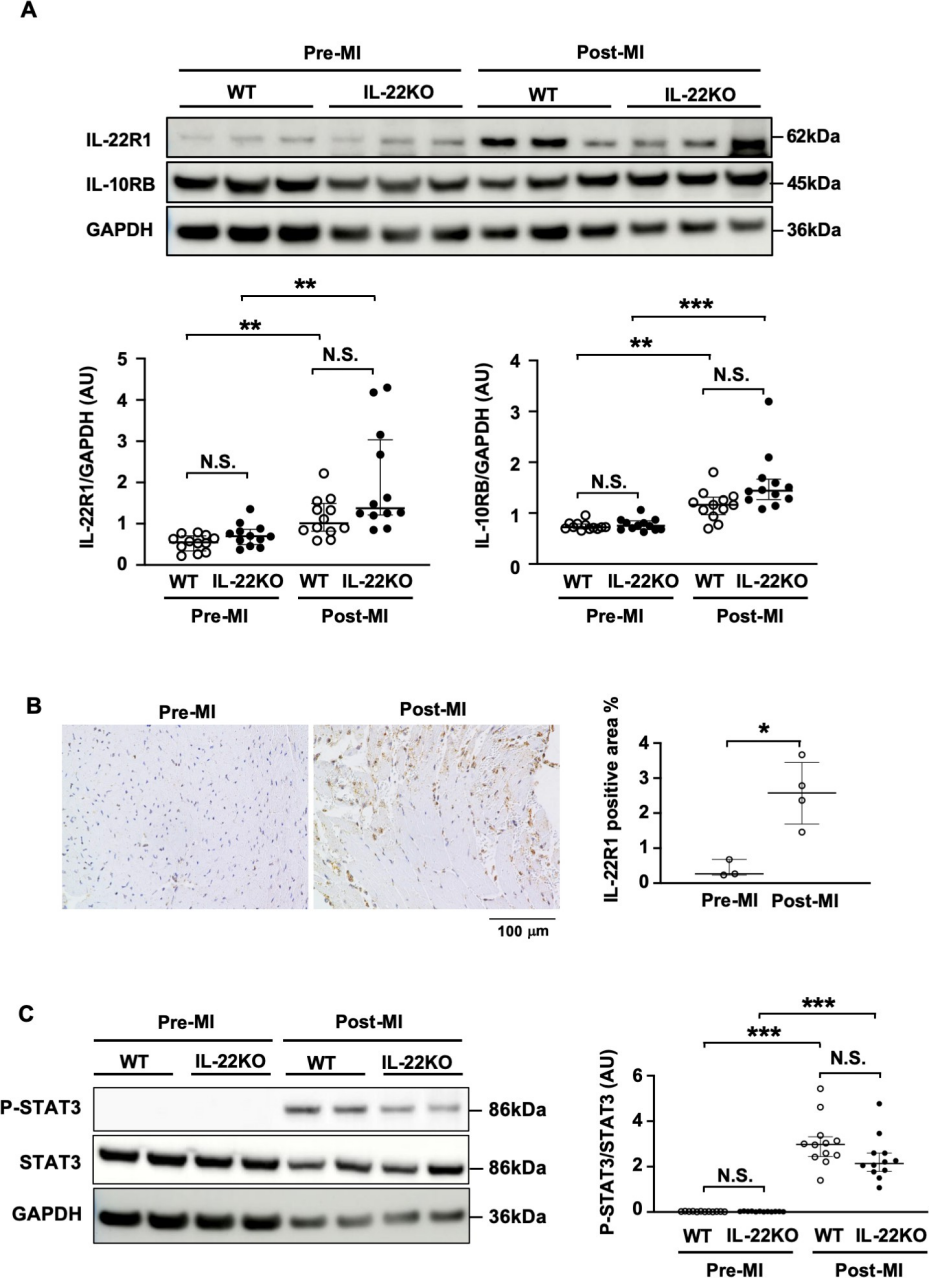
Expression of IL-22 receptor and STAT3 activation in heart tissues after MI. (**A**) Total lysates were prepared from whole ventricle before MI or infarct area 3 days after MI in WT and IL-22KO mice. Representative images are shown for Western blot for IL-22R1, IL-10RB and GAPDH. Graphs show the ratio of IL-22R1 or IL-10RB to GAPDH (n = 12 for each group); *P < 0.05 vs. Pre-MI, Kruskal-Wallis test/Dunn’s multiple comparison test. (**B**) Representative images and quantitative analysis of immunostaining for IL-22R1 in the hearts from WT mice before and 3 days after MI. Values are expressed as the percentage of IL-22R1-positive area per tissue area (n = 3–4 for each group); *P < 0.05 vs. Pre-MI, Mann-Whitney test. (**C**) Total lysates were prepared from whole ventricle before MI or infarct area 3 days after MI. Representative images are shown for the Western blots of p-STAT3, STAT3 and GAPDH. Graphs show the ratio of p-STAT3 to STAT3 (n = 10 for each group); ***P < 0.001, N.S., non-significant, one-way ANOVA/Dunnett’s T3 multiple comparison test.

We examined IL-22 levels in serum and IL-22 gene expression in the heart before and after MI. There was no significant change in serum IL-22 after MI or sham operation compared to that before MI in WT mice **(S2A and S2B Fig)**. RT-PCR showed no change in IL-22 expression in the heart after MI compared to before MI **(S2C Fig)**. IL-22 was not detected in neither blood nor heart in IL-22KO mice. We also examined the protein expression of IL-22 receptor subunits, IL-22R1 and IL-10RB, before and after MI. Western blot analysis revealed that expression of IL-22R1 and IL-10RB was significantly higher in the heart 3 days after MI compared to those before MI regardless of the genotype **(Fig 7A)**. Sham-operated WT mice showed no significant increase in IL-22R1 or IL-10RB compared to before MI **(S2D Fig).** Immunostaining showed that IL-22R1-positive cells were significantly increased in the heart 3 days after MI compared to those before MI **(Fig 7B)**. After MI, STAT3 activation (phosphorylation) was significantly increased compared to that before MI, regardless of the genotype **(Fig 7C)**. We found no significant difference in STAT3 activation between WT mice and IL-22KO mice after MI.

### Effect of IL-22 administration on left ventricular remodeling after MI in IL-22KO mice

Next we evaluated the effect of administration of recombinant IL-22 on left ventricular remodeling after MI in IL-22KO mice. After a single IL-22 administration, serum IL-22 levels at 6 hours were comparable to those of WT mice and at the detection limit at 12 hours **(S3 Fig)**. We administered recombinant IL-22 twice daily to maintain detectable levels of IL-22 in the serum of IL-22KO mice. The levels of activated (phosphorylated) STAT3 was not altered by the IL-22 administration in cardiac tissue. Cardiac rupture after MI did not occur neither in the recombinant IL-22-treated group nor in the PBS group in this set of experiment. Echocardiographic study showed that the left ventricular geometry and function were comparable between the IL-22-treated group and the PBS group on 7 and 14 days after MI **(S4 Fig)**.

## Discussion

In the current study, we found that IL-22KO mice showed larger infarct area and higher mortality due to cardiac rupture after MI, compared to WT mice. IL-22KO mice exhibited higher number of macrophages and myofibroblasts in the border area of the infarcted heart, and higher expression of TNFα in myocardium after MI. In addition, IL-22KO mice showed higher expression of MMP-2, MMP-9 and Col1a1, and lower expression of TIMP-3 before MI. On the other hand, the left ventricular geometry or function did not differ between WT and IL-22KO mice before or after MI. These findings indicated that endogenous IL-22 plays an important role in reducing the infarct size and cardiac rupture without a major impact on the left ventricular remodeling and function after MI. IL-22 may also be involved in the homeostasis of ECM metabolism before MI.

There are several potential mechanisms for the prevention of cardiac rupture by IL-22. Reduction in infarct size by IL-22, as shown by the larger infarct size in IL-22KO mice, may explain why IL-22KO mice showed higher incidence of cardiac rupture, because infarct size is a major determinant of cardiac rupture [26]. The cardioprotective effect of IL-22 may be mediated by suppression of inflammation as shown by the increased expression of TNF-α and macrophage infiltration in IL-22KO mice, or by improved survival of the damaged cardiomyocytes by IL-22 as reported previously [17]. Another possibility is that IL-22 may support the synthesis of ECM and reinforcement of the damaged tissue, as suggested by the less significant expression of Col3a1 after MI in IL-22KO mice. Paradoxically, IL-22KO mice showed more infiltration of F061SMA-positive myofibroblasts which represents activated fibroblasts that can synthesize ECM during healing process after MI [27]. These findings suggest that the myofibroblasts in IL-22KO mouse heart after MI may be dysfunctional with regard to the ECM synthesis and tissue reinforcement. Consistent with this notion, IL-22 has been reported to promote the differentiation of smooth muscle cells and fibroblasts into ECM secretory phenotypes [28, 29]. Yet another possibility is that IL-22 may play an important role in maintaining the homeostasis of ECM metabolism before MI, as the absence of IL-22 caused the imbalance in MMPs and TIMP-3, an endogenous inhibitor of MMPs, before MI. Although histological observation of IL-22KO mouse hearts showed no gross abnormality, it is possible that microarchitecture of ECM may differ between WT and IL-22KO hearts. The abnormal ECM metabolism in the heart of IL-22KO mice, either due to the imbalance in MMPs/TIMP-3 before MI or due to the dysfunction of myofibroblasts after MI, may be reflected to the distorted expression pattern of ECM-related genes as demonstrated by the PCR array data after MI in this study. Multiple roles of IL-22 have been proposed in other organs including skin and intestine, where IL-22 plays important roles both in maintaining healthy condition and in disease conditions such as wound repair [6, 7]. Considering the multiple roles of IL-22, it may prevent cardiac rupture after MI through multiple mechanisms as discussed above.

We found that serum concentration of IL-22 did not change before and after MI, consistent with a previous report [16]. On the other hand, the expression levels of IL-22R1 and IL-10RB, components of IL-22 receptor complex to activate STAT3, was increased after MI. This finding is consistent with recent reports that described the increase in IL-22R1 expression in the heart in pathological conditions, such as cardiac hypertrophy [30] and myocarditis [31, 32]. We found that the P-STAT3/STAT3 ratio was increased in cardiac tissue after MI, regardless of the genotype. While the P-STAT3/STAT3 ratio tended to be lower in IL-22KO than in WT mice, the difference did not reach statistical significance. Because MI induces multiple STAT3-activating cytokines, including IL-6, erythropoietin, and granulocyte-colony stimulating factor, the STAT3-activating effect of endogenous IL-22 may have been masked by other cytokines. The effect of IL-22 is conveyed through its binding to receptor complex consisting of IL-10RB, a common subunit of IL-10 family receptors, and IL-22R1, a subunit specific for IL-22, to activate STAT3 [11, 13, 33]. Therefore, the effect of IL-22 after MI is likely mediated by the enhanced expression of IL-22R1/IL-10RB in cardiac tissue. Because IL-22R1, which determines the receptor specificity for IL-22, is expressed in non-immune cells including epithelial cells, fibroblasts, endothelial cells [11–13], and cardiomyocytes [17], endogenous IL-22 would exert its cardioprotective effect through the function of these cell types after MI. Alternatively, cardioprotection by IL-22 may be exerted by the maintenance of tissue homeostasis before MI, as discussed above, through the IL-22R1/IL-10RB expression at low levels in non-immune cells.

Despite the larger infarct size in IL-22KO mice than in WT mice, the left ventricular geometry or function after MI did not show significant difference except for the interventricular septal thickness at day 5. This discrepancy between the infarct size and the function may be because the difference in the infarct size between WT and IL-22KO mice was not large enough to cause the difference in the left ventricular function. Alternatively, the reduced left ventricular function in IL-22KO mice may have been compensated for by the residual myocardium. Overall, these findings indicate that endogenous IL-22 plays an important role in preventing cardiac rupture in the acute phase but its role in the left ventricular remodeling following MI is limited. Administration of recombinant IL-22 to IL-22KO mice did not alter the left ventricular geometry or function after MI, supporting the notion that IL-22 does not influence the left ventricular remodeling after MI. However, IL-22KO mice with PBS administration did not suffer from cardiac rupture after MI, even though the deterioration of left ventricular geometry and function was comparable to that in IL-22KO mice without PBS administration. Therefore, this experimental setting did not allow us to test the acute effect of IL-22 in preventing cardiac rupture, which needs to be explored in future studies. The absence of cardiac rupture in IL-22KO mice with PBS administration, as opposed to the high rate of cardiac rupture in those without PBS administration, may be caused by the different experimental procedure including the subcutaneous injection of PBS.

## Conclusion

We propose endogenous IL-22 is important in preventing cardiac rupture but not in left ventricular remodeling after MI. The cardioprotective effect of endogenous IL-22 may be mediated by regulation of inflammatory response including myofibroblast activation after MI, or by the maintaining ECM homeostasis before MI. Further study is required to clarify when and on which cell types IL-22 works to protect myocardium from rupture after MI.

## Supporting information

S1 Fig Effect of IL-22 gene deletion on the expression of IL-10 family cytokine. Total RNA was prepared from whole ventricle before MI or infarct heart 3 days after MI and subjected to real-time PCR analysis. Values are normalized to *Gapdh* and expressed as the fold change from the values in WT mice before MI (n = 5 to 7 for each group); *P < 0.05, **P < 0.01, N.S., non-significant, Kruskal-Wallis test/Dunn’s multiple comparison test.
(TIF)

S2 Fig IL-22 and IL-22 receptor expression after MI and sham. Serum levels of IL-22 at the indicated time points before and after MI (A) and sham(B) were determined by microbeads-based assay (n = 3 to 8 for each group). *P < 0.05 vs before MI. Kruskal-Wallis test/Dunn’s multiple comparison test. (C) Total RNA was prepared from whole ventricle before and after MI and subjected to real-time PCR analysis. Values are normalized to *Gapdh* and expressed as the fold change from the values in WT mice before MI (n = 2 to 7 for each group); N.S., non-significant, Kruskal-Wallis test/Dunn’s multiple comparison test. (D) Total cell lysates from WT mice were prepared from whole ventricle before MI, the infarct heart or left ventricle of sham 3 days after MI. Blots were probed using antibodies against IL-22R1, IL-10RB and GAPDH. Graphs represent quantitative differences in expression based on the ratio of IL-22R1 to GAPDH or IL-10RB to GAPDH (n = 3 for each group); *P < 0.05 vs. Pre-MI, Kruskal-Wallis test/Dunn’s multiple comparison test.
(TIF)

S3 Fig Changes in blood IL-22 levels and cardiac STAT3 activation by recombinant IL-22 administration. (A) Serum levels of IL-22 at the indicated time points after the administration of recombinant IL-22 (n = 3 to 8 for each group). N.S., non-significant, Kruskal-Wallis test/Dunn’s multiple comparison test. (B) Total cell lysates were prepared from whole ventricle of IL-22KO mice at the indicated time points after the recombinant IL-22 administration. Representative images are shown for the Western blots of p-STAT3, STAT3 and GAPDH. Graphs show the ratio of p-STAT3 to STAT3 (n = 3 for each group); *P < 0.05, Kruskal-Wallis test/Dunn’s multiple comparison test.
(TIF)

S4 Fig Effects of recombinant IL-22 administration on left ventricular geometry and function after MI in IL-22KO mice. Echocardiography was performed at the indicated time points (n = 4–7 for each group); *P < 0.05, N.S., non-significant, Kruskal-Wallis test/Dunn’s multiple comparison test. LVID:d, left ventricular internal dimension in diastole; LVID:s, left ventricular internal dimension in systole; interventricular septal thickness in diastole; IVS:d, left ventricular posterior wall thickness in diastole; LVPW:d, EF, ejection fraction.
(TIF)

S5 Fig Original images of whole membranes. Membranes are shown for western blotting in Figs 7A and 7C and S2D and S3B. Red rectangles indicate the area used in corresponding figures. X indicate lanes not included in figures. MW; molecular weight marker.
(TIF)
