# Supplementary figures and images for: Endogenous interleukin-22 prevents cardiac rupture after myocardial infarction in mice
Source: PLoS One. 2023 Jun 15;18(6):e0286907. doi: 10.1371/journal.pone.0286907 (PMC10270598; doi:10.1371/journal.pone.0286907)

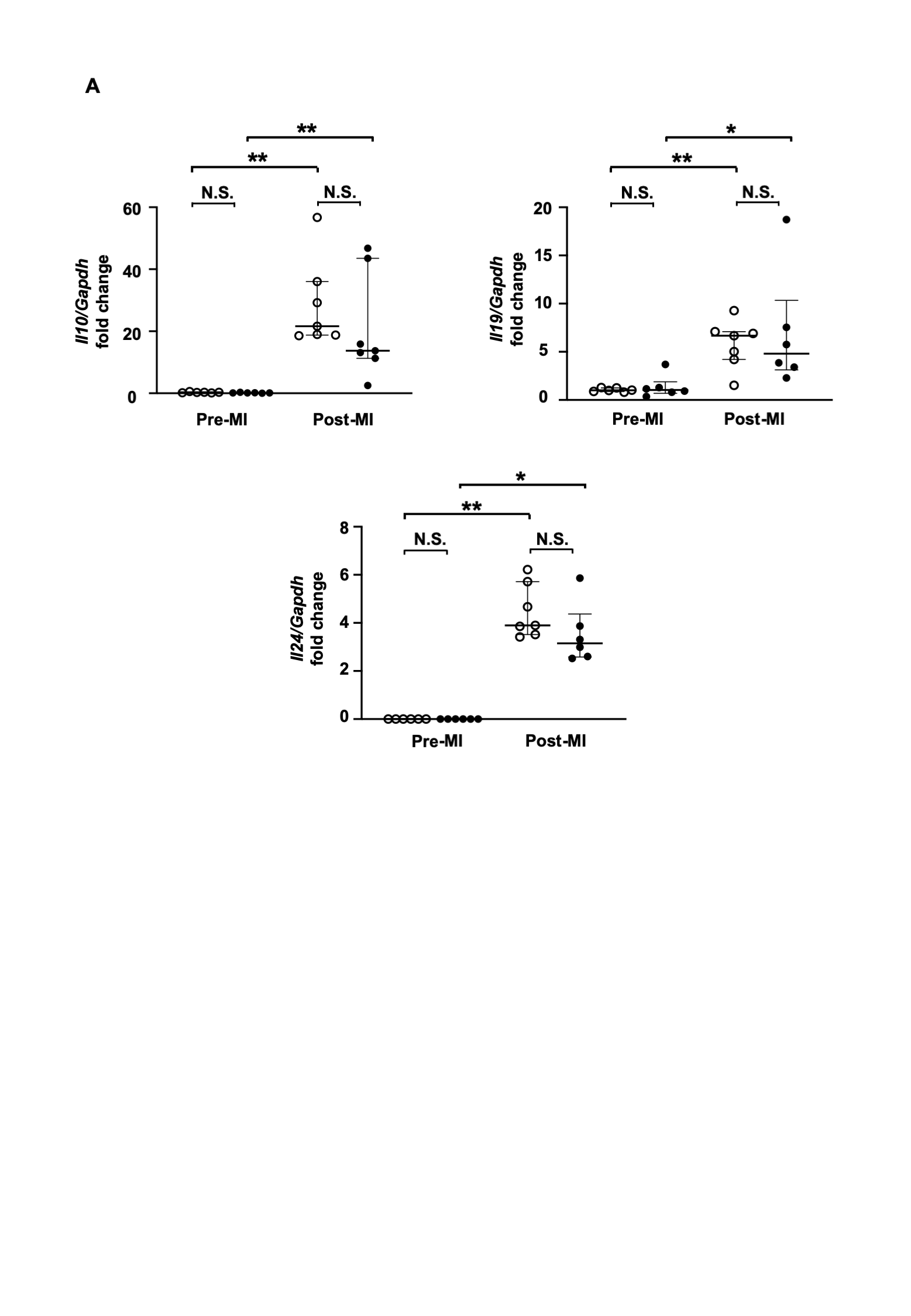

Supplement: S1 Fig — Total RNA was prepared from whole ventricle before MI or infarct heart 3 days after MI and subjected to real-time PCR analysis. Values are normalized to Gapdh and expressed as the fold change from the values in WT mice before MI (n = 5 to 7 for each group); *P < 0.05, **P < 0.01, N.S., non-significant, Kruskal-Wallis test/Dunn’s multiple comparison test. (TIF) [file pone.0286907.s001.tif]

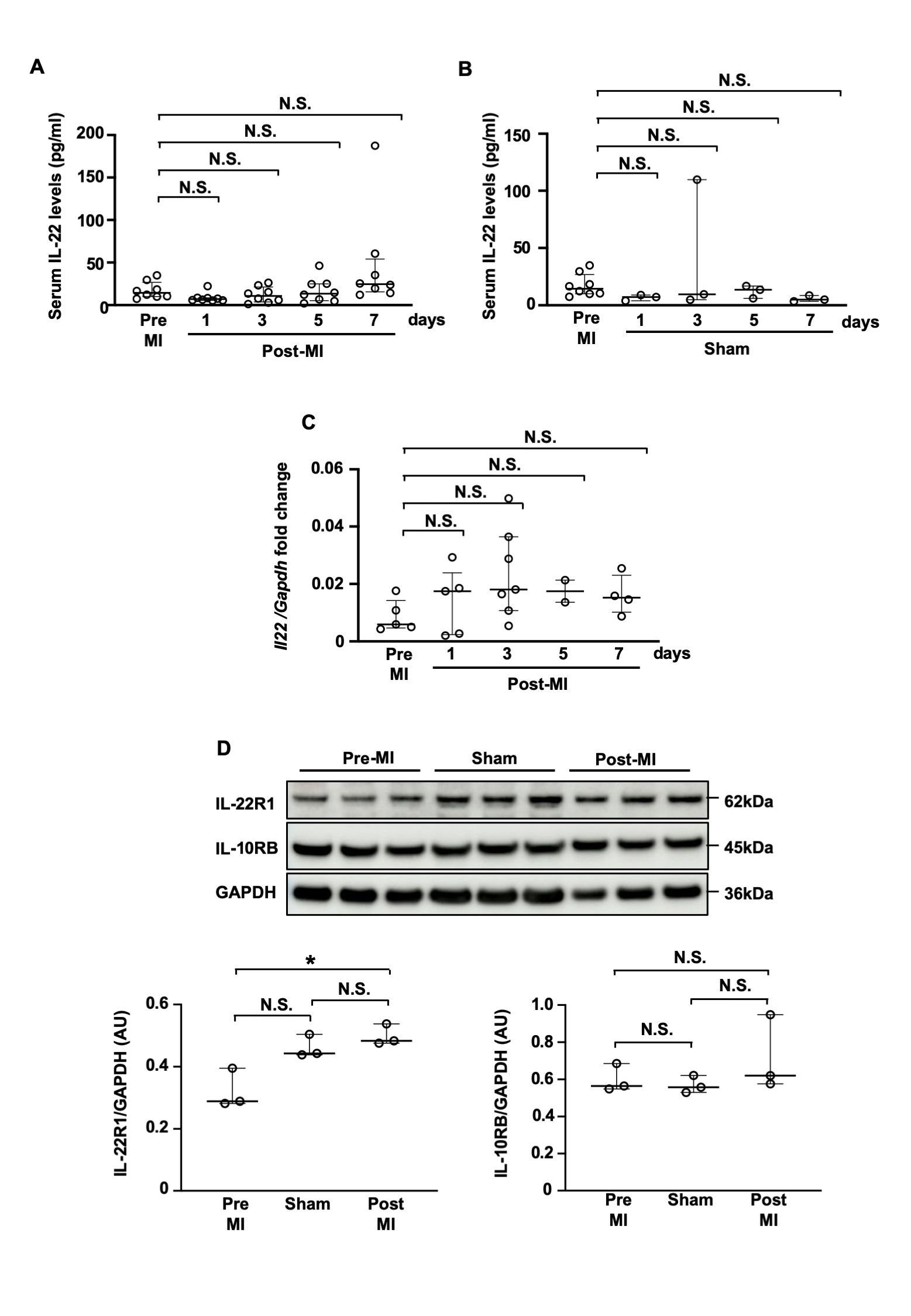

Supplement: S2 Fig — Serum levels of IL-22 at the indicated time points before and after MI (A) and sham(B) were determined by microbeads-based assay (n = 3 to 8 for each group). *P < 0.05 vs before MI. Kruskal-Wallis test/Dunn’s multiple comparison test. (C) Total RNA was prepared from whole ventricle before and after MI and subjected to real-time PCR analysis. Values are normalized to Gapdh and expressed as the fold change from the values in WT mice before MI (n = 2 to 7 for each group); N.S., non-significant, Kruskal-Wallis test/Dunn’s multiple comparison test. (D) Total cell lysates from WT mice were prepared from whole ventricle before MI, the infarct heart or left ventricle of sham 3 days after MI. Blots were probed using antibodies against IL-22R1, IL-10RB and GAPDH. Graphs represent quantitative differences in expression based on the ratio of IL-22R1 to GAPDH or IL-10RB to GAPDH (n = 3 for each group); *P < 0.05 vs. Pre-MI, Kruskal-Wallis test/Dunn’s multiple comparison test. (TIF) [file pone.0286907.s002.tif]

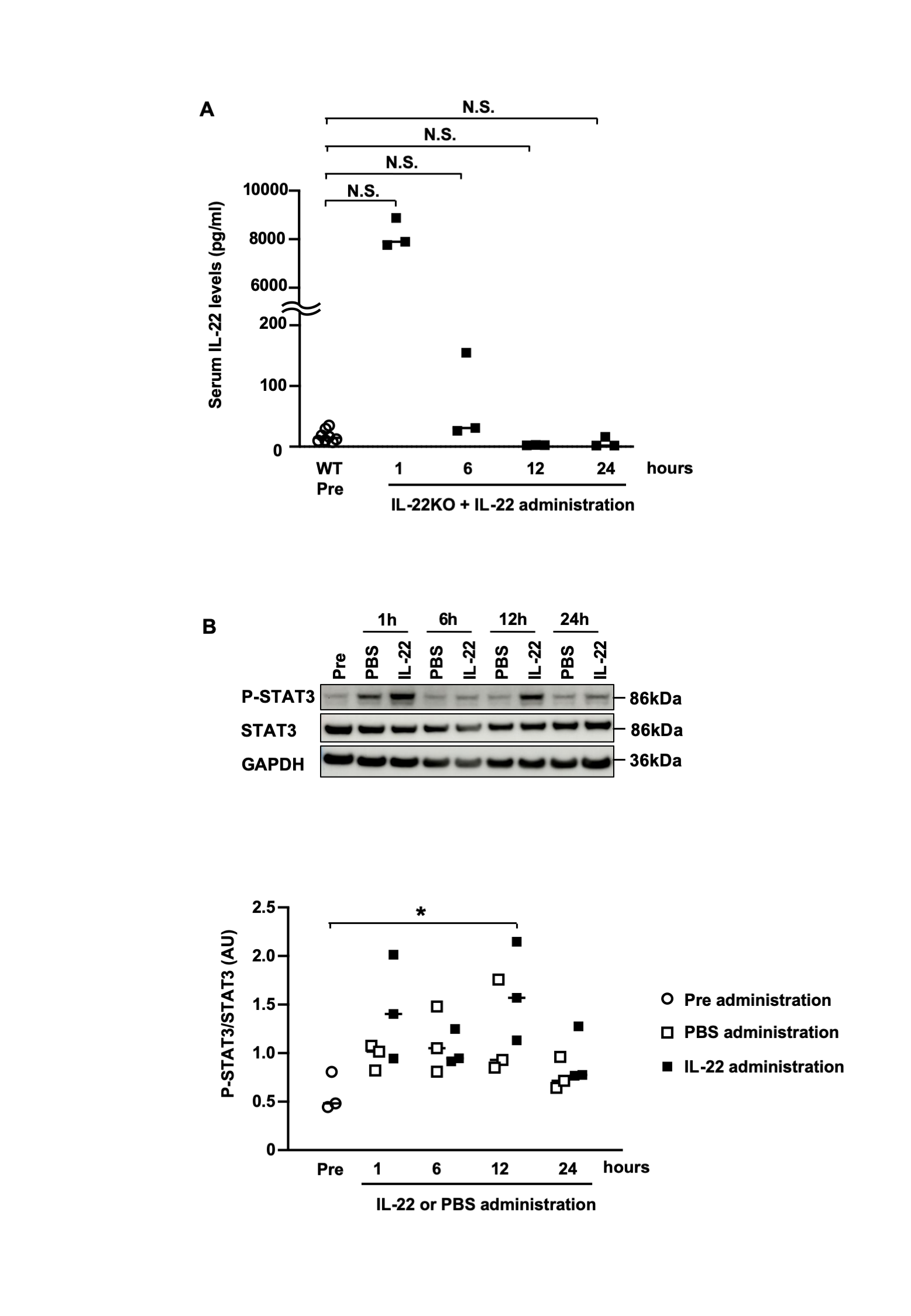

Supplement: S3 Fig — (A) Serum levels of IL-22 at the indicated time points after the administration of recombinant IL-22 (n = 3 to 8 for each group). N.S., non-significant, Kruskal-Wallis test/Dunn’s multiple comparison test. (B) Total cell lysates were prepared from whole ventricle of IL-22KO mice at the indicated time points after the recombinant IL-22 administration. Representative images are shown for the Western blots of p-STAT3, STAT3 and GAPDH. Graphs show the ratio of p-STAT3 to STAT3 (n = 3 for each group); *P < 0.05, Kruskal-Wallis test/Dunn’s multiple comparison test. (TIF) [file pone.0286907.s003.tif]

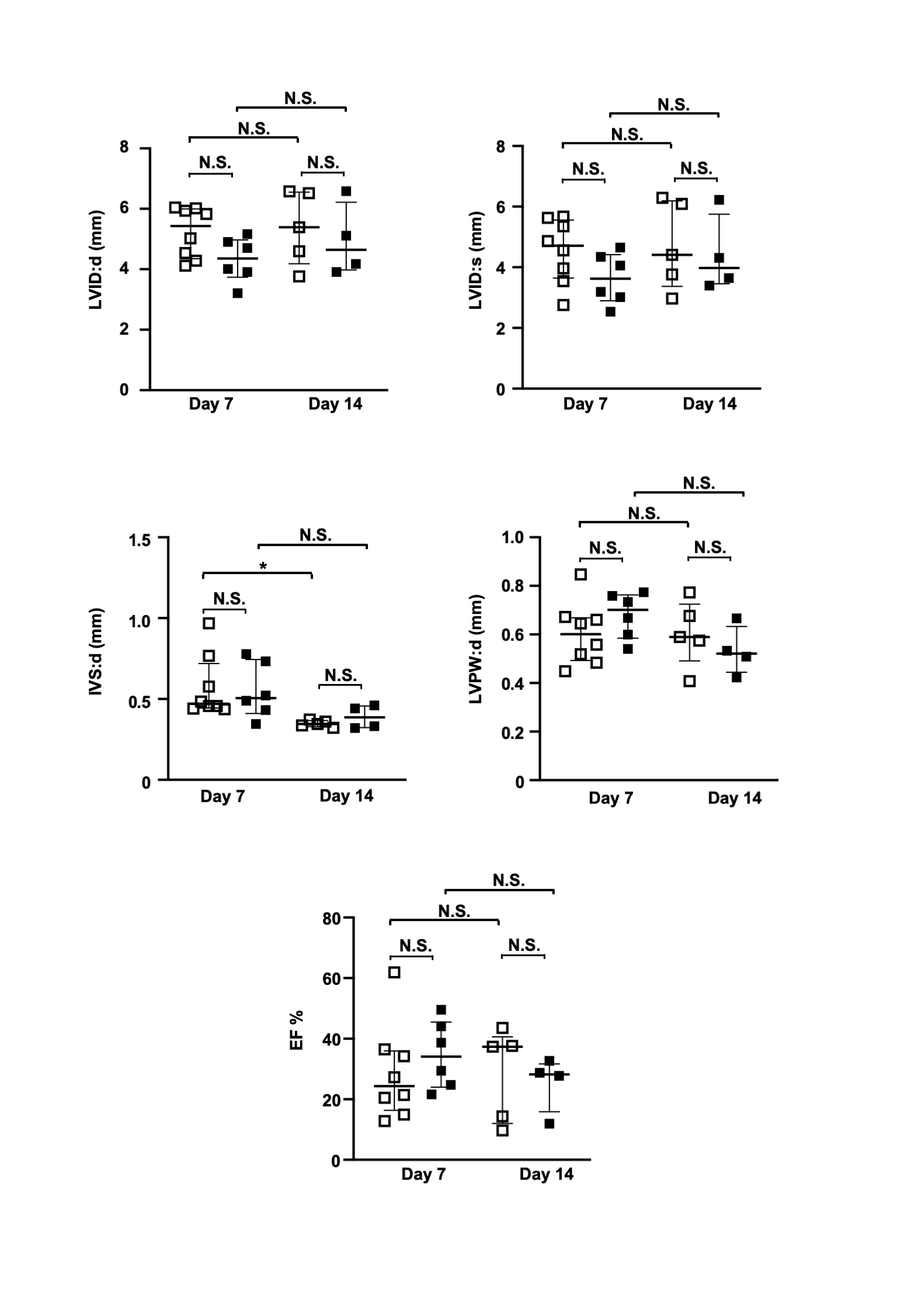

Supplement: S4 Fig — Echocardiography was performed at the indicated time points (n = 4–7 for each group); *P < 0.05, N.S., non-significant, Kruskal-Wallis test/Dunn’s multiple comparison test. LVID:d, left ventricular internal dimension in diastole; LVID:s, left ventricular internal dimension in systole; interventricular septal thickness in diastole; IVS:d, left ventricular posterior wall thickness in diastole; LVPW:d, EF, ejection fraction. (TIF) [file pone.0286907.s004.tif]

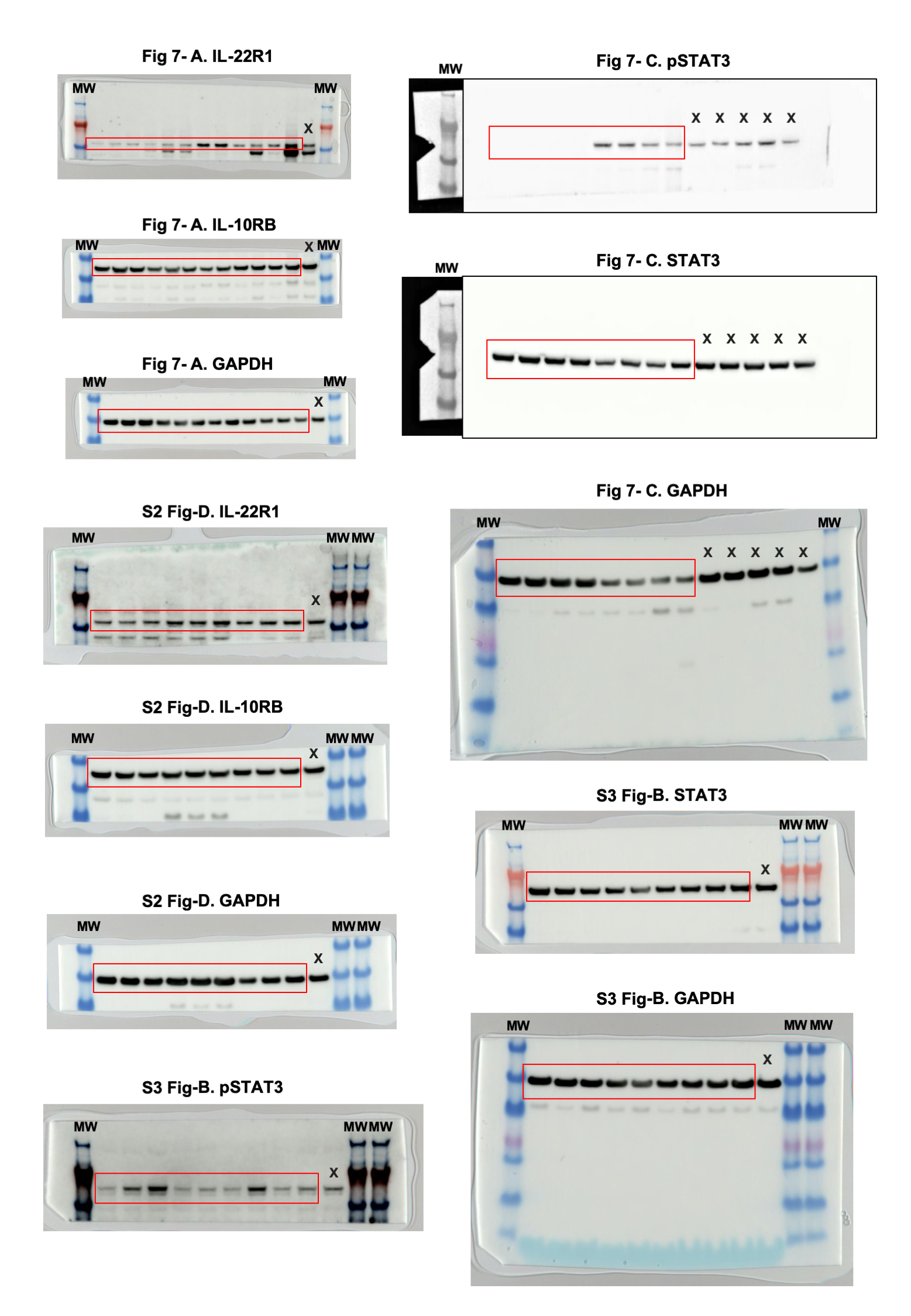

Supplement: S5 Fig — Membranes are shown for western blotting in Figs 7A and 7C and S2D and S3B. Red rectangles indicate the area used in corresponding figures. X indicate lanes not included in figures. MW; molecular weight marker. (TIF) [file pone.0286907.s005.tif]
